# Supplementary material for: Year-Long Microbial Succession on Microplastics in Wastewater: Chaotic Dynamics Outweigh Preferential Growth
Source: Microorganisms. 2022 Sep 2;10(9):1775. doi: 10.3390/microorganisms10091775 (PMC9506493; doi:10.3390/microorganisms10091775)
Supplement: Supplementary file 1 [file microorganisms-10-01775-s001.zip › microorganisms-1886676-supplementary.pdf]

Year-Long Microbial Succession on Microplastics in Wastewater: Chaotic Dynamics Outweigh Preferential Growth

Alexander S. Tagg [1,2,*], Theodor Sperlea [1], Matthias Labrenz [1], Jesse P. Harrison [3], Jesús J. Ojeda [2] and Melanie Sapp [4]

[1] Leibniz-Institut für Ostseeforschung Warnemünde, Seestraße 15, 18119 Rostock, Germany
[2] Department of Chemical Engineering, Faculty of Science and Engineering, Swansea University, Swansea SA1 8EN, UK
[3] CSC—IT Center for Science Ltd., P.O. Box 405, FI-02101 Espoo, Finland
[4] Institute of Human Genetics, University Hospital Düsseldorf, Heinrich Heine University, Moorenstrasse 5, 40225 Düsseldorf, Germany
* Correspondence: alexandertagg@gmail.com

Experimental Section

Microplastic Information

The microplastic types studied were PE (Goodfellow Ltd, Cambridge, UK), PP (Shawingigan Ltd, London, UK), PVC (Cellomer Associates inc., New York, USA), nylon-6 (Koch-Light Laboratories Ltd, Colnbrook, UK) and trimmed PET fragments obtained from a commercial mineral water bottle (Costcutter Montgomery Q4 Natural Water, Uxbridge, UK). With the exception of PET, all microplastics were ~1.

Microplastic Extraction from Microcosm Tubes

Larger microplastics (e.g. PET) and large glass beads were removed at each sampling interval using a small spatula. Smaller microplastics (150–250 µm) were extracted by multiple pipetting and rinsing with sterilised ultra-high quality [UHQ] water. This was repeated 3–6 times until no more biogenic organic matter (BOM) could be observed. In a final step all microplastics were drawn into a 250-µL pipette tip and the remaining liquid was absorbed using sterilised tissue paper (VWR, Germany). The outside of the tip was cleaned with 80% (*v*/*v*) ethanol (VWR, Germany) and the retained microplastics were stored in 1 ml of UHQ water at 4 °C until all samples were ready for DNA extraction. All microplastic extraction work was undertaken in a laminar flow cabinet to avoid contamination from the air.

For microplastics which would form a pellet by centrifugation, samples were centrifuged (up to 2038× *g*) for 2 minutes before being extracted from storage suspension. The sample was placed on clean, water resistant laboratory film (Parafilm MTM, Sigma Aldrich, USA) and the remaining suspension liquid was removed using a fine 2 µL pipette tip (see Appendix B; Figure B1). For PE and PP, which could not be centrifuged, a 1 µL inoculation loop was used to transfer microplastics from storage suspension directly to the extraction tube. For the extraction of larger size microplastics like PET and large glass beads (~1–2 mm) sterile fine tweezers were used for transfer. Glass microbeads were transferred to extraction tubes using a 20 µL pipette tip without ejecting any of the suspension fluid.

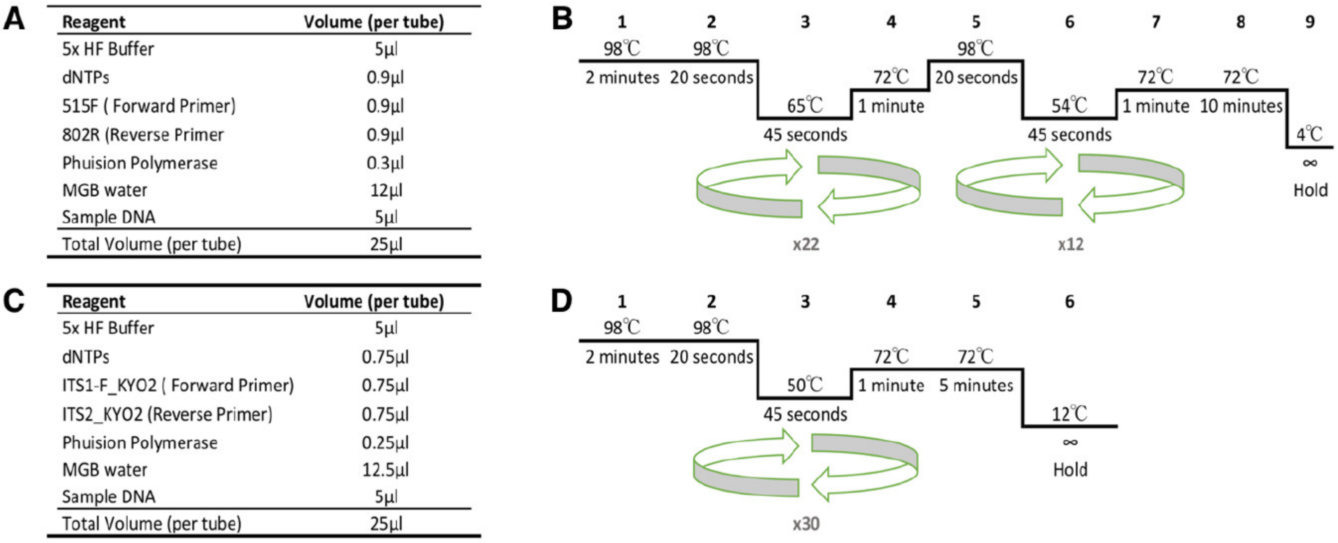

A

Reagent	Volume (per tube)
5x HF Buffer	5μl
dNTPs	0.9μl
515F (Forward Primer)	0.9μl
802R (Reverse Primer	0.9μl
Phuision Polymerase	0.3μl
MGB water	12μl
Sample DNA	5μl
Total Volume (per tube)	25μl

C

Reagent	Volume (per tube)
5x HF Buffer	5μl
dNTPs	0.75μl
ITS1-F_KYO2 (Forward Primer)	0.75μl
ITS2_KYO2 (Reverse Primer)	0.75μl
Phuision Polymerase	0.25μl
MGB water	12.5μl
Sample DNA	5μl
Total Volume (per tube)	25μl

Figure S1. Experimental details of amplicon PCRs. (**A**): Amplicon PCR mix for 16S. (**B**): Amplicon PCR run protocol for 16S. (**C**): Amplicon PCR mix for ITS. (**D**): Amplicon PCR run protocol for ITS.

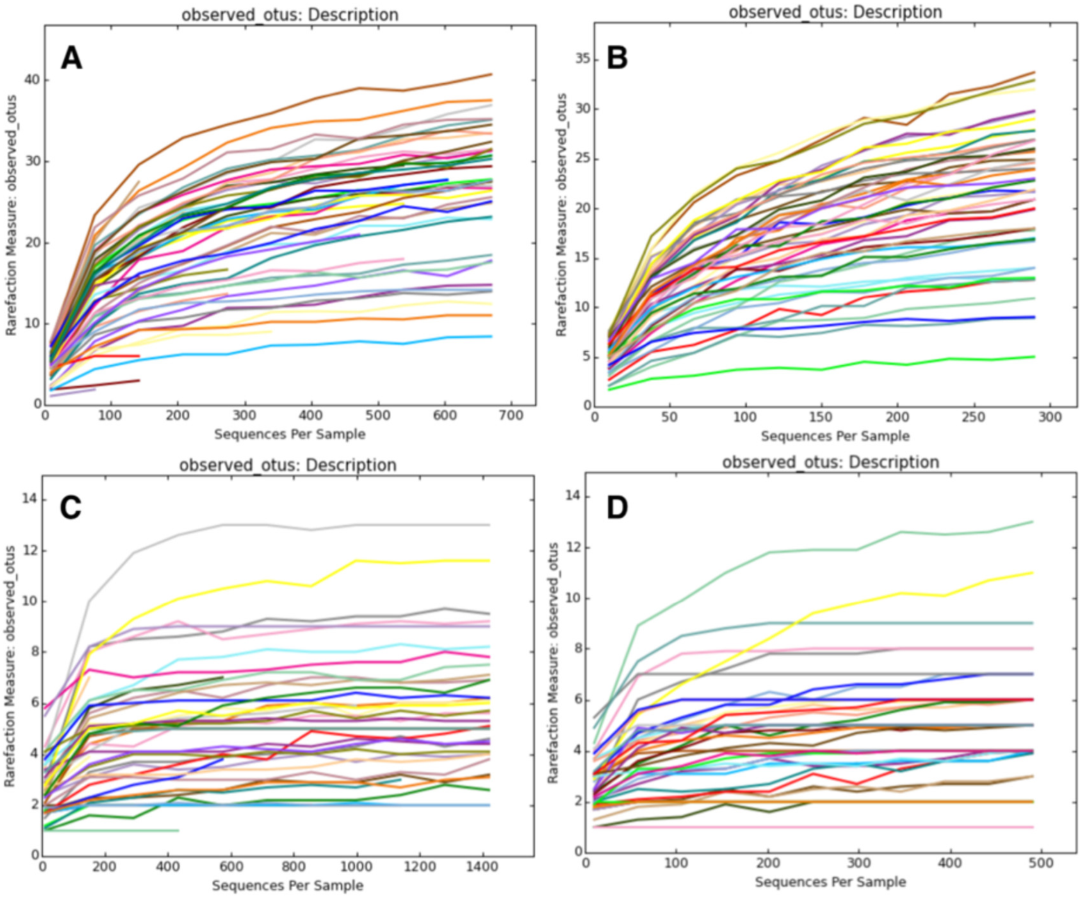

Figure S2. Rarefication curves based on OTU richness. A: 16S before rarefication; B: 16S after rarefication at 297 sequences per sample; C: ITS before rarefication; D: ITS after rarefication at 499 sequences per sample.

Table S1. National Centre for Biotechnology Information (NCBI) Basic Local Alignment Search Tool (BLAST) best matches for 16S OTUs. See main text for full references.

OTU ID	UTAX Taxonomy	BLAST "Best Hit" Taxonomy	BLAST Score	BLAST Similarity (%)	"Best Hit" Reference (If Available)	"Best Hit" Genbank ID
OTU 3	*Pseudomonas sp.*	*Pseudomonas* sp.	468	100	Hamm et al., 2017 [78]	KX928315
OTU 7	*Rhodanobacter A*	*Rhodonobacter thiooxyidans*	468	100	Thijs et al., 2014 [79]	HG794331
OTU 8	*Armatimonas Gp1*	Uncultured bacterium	470	100	Unpublished	KU113498
OTU 10	*Sphingomonas*	*Sphingomonas* sp.	468	100	Colin et al., 2017 [80]	KX419227
OTU 11	*Corynebacterineae*	*Rhodococcus erythropolis*	468	100	Mikolasch et al., 2016 [81]	KU663058
OTU 12	*Mucilaginibacter A*	*Mucilaginibacter sp.*	468	100	Unpublished	EU423305
OTU 14	*Geothrix*	Uncultured bacterium	468	100	Rodrigues et al., 2010 [82]	GU169059
OTU 15	*Dokdonella*	Uncultured bacterium	468	100	Unpublished	KU316012
OTU 16	*Thiobacillus*	Uncultured *Thiobacillus*	468	100	Suzuki et al., 2016 [83]	LC070284
OTU 17	*Mucilaginibacter B*	Uncultured *Bacteroidetes*	468	100	Zinger et al., 2009 [84]	FJ569393
OTU 18	*Rhodanobacter B*	Uncultured bacterium	468	100	Zeglin et al., 2016 [85]	KP909173
OTU 19	*Corynebacterineae*	*Mycobacterium fortuitum*	468	100	Azadi et al., 2016 [70]	KX954379
OTU 20	*Rhodanobacter C*	Uncultured *Rhodanobacter* sp.	468	100	Unpublished	KY445711
OTU 21	*Unidenitifed Edaphobacter (Order)*	Uncultured bacterium	468	100	Zeglin et al., 2016 [85]	KP911732
OTU 22	*Micrococcineae*	*Lysinimonas* sp.	468	100	Liu et al., 2002 [86]	KT803390
OTU 23	*Povalibacter*	Uncultured bacterium	468	100	Peipoch et al., 2015 [87]	KT908136
OTU 24	Unidenitifed *Rhizomicrobium* (Family)	Uncultured bacterium	468	100	Zeglin et al., 2016 [85]	KP911577
OTU 25	*Devosia*	*Devosia* sp.	468	100	Colin et al., 2017 [80]	KX418822
OTU 26	*Acidocella*	Uncultured *Acidoella* sp.	468	100	Unpublished	EF016441
OTU 28	*Chitinophaga*	Uncultured *Bacteroidetes*	468	100	Zinger et al., 2009 [84]	FJ569783
OTU 29	Unidentified *Terriglobus* (Order)	Uncultured *Granulicella* sp.	468	100	Unpublished	KR831665
OTU 30	*Afipia*	*Nitrobacter* sp.	468	100	Unpublished	KY117526
OTU 32	*Flavisolibacter*	Uncultured bacterium	468	100	Fonseca-García et al., 2016 [88]	KU539980
OTU 33	*Ferruginibacter*	Uncultured *Sphingobacteriales bacterium*	462	99	Sun et al., 2012 [89]	JN541173
OTU 35	*Aminobacter*	*Mesorhizobium ciceri*	468	100	Haskett et al., 2016 [71]	KX226352
OTU 36	*Burkholderia*	*Burkholderia sp.*	468	100	Colin et al., 2017 [80]	KX419240
OTU 38	*Pseudonocardineae*	*Pseudonocardia xishanensis*	470	100	Zhao et al., 2012 [90]	NR_108411
OTU 39	*Mesorhizobium*	*Mesorhizobium waimense*	468	100	De Meyer et al., 2015 [91]	NR_137372
OTU 40	Unidenitifed *Aridibacter* (Order)	Uncultured bacterium	462	99	Yin et al., 2015 [92]	KP784910
OTU 41	*Brevundimonas*	Uncultured *alpha proteobacterium*	468	100	Yergeau et al., 2007 [93]	EF219826
OTU 42	*Nevskia*	Uncultured bacterium	468	100	Zeglin et al., 2016 [85]	KP912254
OTU 43	Unidenitifed *Verrucomicrobia* Sub3 (Class)	Uncultured bacterium	468	100	Dunbar et al., 2012 [94]	JQ366564
OTU 45	*Oleiagrimonas*	Uncultured archaeon	468	100	Rastogi et al., 2009 [95]	FJ184749
OTU 46	*Beijerinckia*	Uncultured *Rhizobiales*	468	99	Hodkinson et al., 2012 [96]	JF814502
OTU 47	*Rhodopseudomonas A*	Uncultured bacterium	468	100	Kelly et al., 2010 [97]	GU219805
OTU 52	*Flavihumibacter*	Uncultured *Bacteroidetes*	468	100	Zinger et al., 2009 [84]	FJ569311
OTU 53	*Romboutsia*	*Romboutsia timonensis*	466	100	Ricaboni et al., 2016 [98]	NR_1447
OTU 54	*Phenylobacterium*	Uncultured bacterium	462	99	Zeglin et al., 2016 [85]	KP904793
OTU 55	*Nitrobacter*	Uncultured bacterium	468	100	Falteisek and Cepicka, 2012 [99]	JQ217878
OTU 56	Unidentified *Rhizomicrobium* (Family)	Uncultured alpha proteobacterium	468	100	Chen et al., 2014 [100]	KF182818
OTU 61	*Flavisolibacter*	*Flavitalea* sp.	468	100	Unpublished	KX146487
OTU 62	WPS-1 "*Genera incertae sedis*" (Class)	Uncultured bacterium	446	98	Unpublished	JQ711725
OTU 63	Unidentified *Acidobacteria* Gp1 (Class)	Uncultured bacterium	451	99	Zeglin et al., 2016 [85]	KP913651
OTU 68	*Parvibaculum*	Sediment bacterium	468	100	Hilyard et al., 2008 [101]	EU167984
OTU 69	*Rhodopseudomonas B*	*Rhodopseudomonas* sp.	468	100	Ganzert et al., 2014 [102]	KF974286
OTU 73	*Acidicaldus*	Uncultured bacterium	462	99	Zeglin et al., 2016 [85]	KP929003
OTU 74	*Aquisphaera B*	Uncultured bacterium	464	99	Fonseca-Garcia et al., 2016 [88]	KU542490
OTU 76	*Micrococcineae*	*Leifsonia lichenia*	468	100	Al-Sadi et al., 2016 [103]	KU220847
OTU 77	Unidentified *Acidobacteria Gp3* (Class)	Uncultured *Acidobacteria*	468	99	Unpublished	KR844218
OTU 78	*Ferruginibacter*	Uncultured bacterium	468	100	Zeglin et al., 2016 [85]	KP906926
OTU 82	*Dyadobacter*	*Dyadobacter* sp.	468	100	Colin et al., 2017 [80]	KX418930
OTU 84	*Vampirovibrio A*	Uncultured *Vampirovibrio* sp.	468	100	Unpublished	KR839383
OTU 85	*Pectobacterium*	*Pectobacterium carotovorum*	468	100	Al-Kharousi et al., 2016 [104]	KR265429

OTU 89	*Lacibacterium*	Uncultured *Rhodospirillaceae*	468	100	Lesaulnier et al., 2008 [105]	EF018478
OTU 92	*Nitrosospira*	Uncultured *Nitrosospira* sp.	468	100	Zhao et al., 2015 [106]	KP890815
OTU 98	*Staphylococcus*	*Staphylococcus epidermidis*	468	100	Sepulveda et al., 2016 [107]	KX108950
OTU 108	*Vampirovibrio B*	Uncultured *Vampirovibrio* sp.	451	99	Unpublished	KR836560
OTU 113	*Haliangiaceae*	Uncultured soil bacterium	468	100	Lueders et al., 2006 [108]	DQ643693
OTU 116	*Pseudolabrys*	Uncultured alpha proteobacterium	468	100	Sun et al., 2012 [89]	JN541152
OTU 129	*Aquisphaera A*	Uncultured bacterium	464	99	Unpublished	KY256905
OTU 136	*Propionibacterineae*	*Marmoricola* sp.	468	100	Unpublished	LN833253
OTU 138	Unidentified *Acidipila* (Order)	Uncultured *Acidobacterium*	468	100	Sanchez-Andrea et al., 2011 [109]	HQ730658
OTU 139	*Candidimonas*	Parapusillimonas granuli	462	99	Auffret et al., 2015 [110]	KM047480
OTU 140	*Ewingella*	*Ewingella* sp.	468	100	Xie et al., 2017 [111]	KX378962
OTU 276	*Herbaspirillum*	*Herbaspirillum hiltneri*	451	99	Poosakkannu et al., 2015 [112]	KJ529095
OTU 373	*Sediminibacterium*	Uncultured bacterium	457	99	Egert et al., 2010 [113]	FN401290
OTU 380	*Mucilaginibacter* C	Uncultured *Bacteroidetes*	451	99	Zinger et al., 2009 [84]	FJ569664
OTU 801	*Mizugakiibacter*	Uncultured *Xanthomonadaceae*	440	98	Ceja-Navarro et al., 2010 [114]	FJ889338

Table S2. National Centre for Biotechnology Information (NCBI) Basic Local Alignment Search Tool (BLAST) best matches for ITS OTUs. See main text for full references.

OTU ID	UTAX Taxonomy	BLAST "Best Hit" Taxonomy	BLAST score	BLAST Similarity (%)	BLAST "Best Hit" Reference (If Available)	"Best Hit" Genbank ID
OTU 1	*Unidentified Tremellomycetes (Class)*	*Trichosporon porosum*	416	99	Jimenez et al., 2014 [115]	KF285994
OTU 2	*Orpinomyces*	*Uncultured fungus*	512	98	Unpublished	KU000514
OTU 3	*Exophiala*	*Exophiala equina*	555	99	Unpublished	JF747078
OTU 5	*Trichoderma*	*Trichoderma lixii*	573	99	Anees et al., 2010 [116]	HM176572
OTU 6	*Candida*	*Candida sake*	366	99	Unpublished	KY106736
OTU 7	*Tyromyces*	*Trypanosoma evansi -Tyropanosome*	124	99	Wen et al., 2016 [117]	KU552351
OTU 10	*Unidentified Nectriaceae (Family)*	*Fusarium oxysporum*	477	100	Unpublished	KT241034
OTU 11	*Unidentified Sordariomycetes (Class)*	*Uncultured fungus*	488	100	Unpublished	JX364633
OTU 13	*Unidentified Fungi (Kingdom)*	*Uncultured eukaryote*	154	98	Unpublished	KY690346
OTU 14	*Serendipita*	*Uncultured fungus*	124	99	Taylor et al., 2008 [118]	EU292563
OTU 15	*Unidentified Basidiomycota (Phylum)*	*Uncultured fungus*	311	91	Unpublished	KU000525
OTU 16	*Unidentified Fungi (Kingdom)*	*Fungal sp. NLEndoHerit_025_2008N1-27-3H*	488	99	Lamit et al., 2014 [119]	JX978254
OTU 17	*Penicillium*	*Penicillium corylophilum*	520	99	Bukovska et al., 2010 [120]	GU566277
OTU 19	*Devriesia*	*Uncultured fungus*	455	100	Adams et al., 2013 [121]	KF221844
OTU 20	*Pulchromyces*	*Metschnikowia matae var. matae*	122	96	De Oliveira Santos et al., 2015 [122]	KR779914
OTU 21	*Pestalotiopsis*	*Pestalotiopsis maculans*	462	99	Unpublished	KX610327
OTU 22	*Unidentified Helotiales (Order)*	*Uncultured fungus*	479	100	Cordier et al., 2012 [123]	JN906781
OTU 23	*Unidentified Nectriacea (Family)*	*Uncultured Ascomycota*	475	99	Kartzinel et al., 2013 [124]	JX998704
OTU 24	*Malassezia*	*Uncultured Malassezia*	671	99	Unpublished	KY430579
OTU 25	*Unidentified Fungi (Kingdom)*	*Uncultured Basidiomycota*	128	99	Unpublished	KU000462
OTU 26	*Guehomyces*	*Tausonia pullulans*	483	99	Unpublished	KY105583
OTU 27	*Unidentified Pleosporales (Order)*	*Alternaria tenuissima*	503	99	Unpublished	KX674653
OTU 28	*Aspergillus*	*Aspergillus pseudoglaucus*	462	99	Unpublished	KX610153
OTU 30	*Unidentified Sporidiobolales (Family Incertae sedis)*	*Sporidiobolales sp. LM531*	479	99	Unpublished	EF060822
OTU 32	*Unidentified Sporidiobolales (Order)*	*Uncultured Sporobolomyces*	479	99	Unpublished	KT334689
OTU 34	*Malassezia*	*Malassezia restricta*	588	99	Alaei et al., 2009 [125]	EU400587
OTU 35	*Unidentified Fungi (Kingdom)*	*Uncultured fungus*	451	89	Scoble et al., 2014 [126]	KF577829
OTU 37	*Sporobolomyces*	*Uncultured fungus*	424	88	Waldrop et al., 2006 [127]	DQ420716
OTU 63	*Monographella*	*Microdochium sp. 00019*	460	99	Carter et al., 1999 [131]	AJ246155
OTU 131	*Unidentified Fungi (Kingdom)*	*Uncultured fungus*	327	96	Unpublished	KU000475
OTU 226	*Unidentified Tremellomycetes (Class)*	*Trichosporon porosum strain 2t1F*	407	99	Jiménez et al., 2012 [115]	KF285994

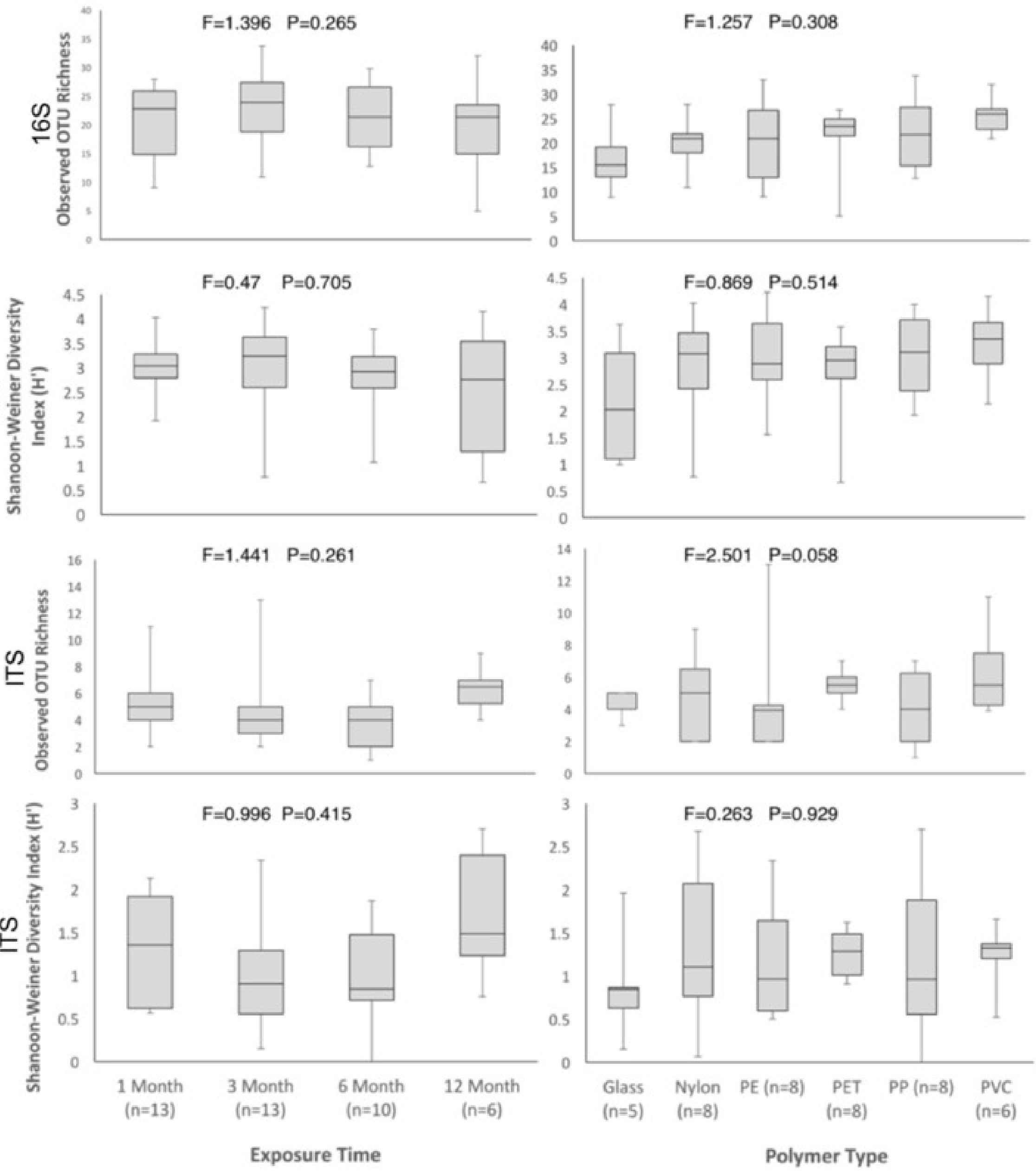

Figure S3. Box plots of bacterial or fungal OTU richness and Shannon-Wiener diversity for both polymer type and time. ANOVA F and *p* values show no significant differences within each tested box plot data (randomly subsampled to highest possible equal level of n). In the case of graph F where the *p* value indicates near-significance, post-hoc Tukey's HSC test proved no significant differences between any variables.

Results

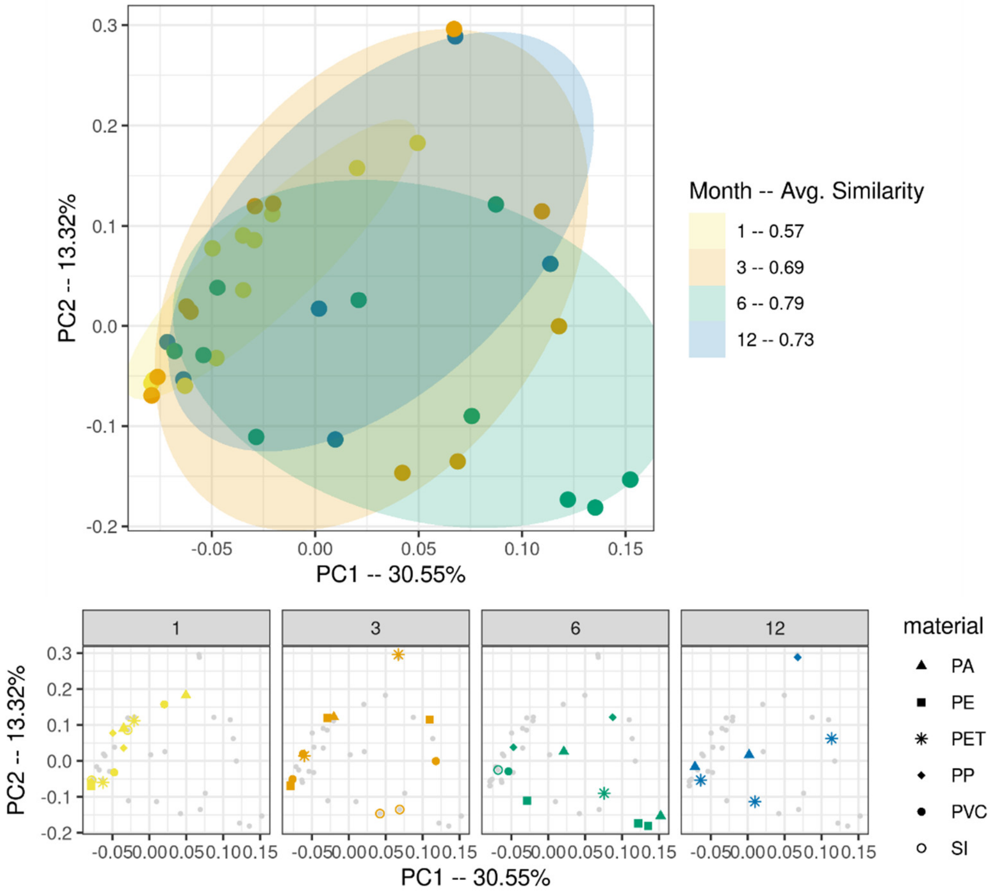

Figure S4. Principle coordinate analyses of ITS data based on Bray-Curtis dissimilarity matrix. The top plot shows the overall dataset while the bottom plots give details on polymer types.
